# Effectiveness and Safety of Combined Semaglutide and Dapagliflozin Therapy in Type 2 Diabetes: A Retrospective Cohort Study

**DOI:** 10.7759/cureus.112523

**Published:** 2026-07-12

**Authors:** Mahmoud Alzahrani, Najlaa Alsudairy, Zahra Al Asmari, Lujainah Basubrain, Hamza Saad, Abdulelah Abdullatif, Zyad Alawashiz, Nabil Alsulami, Muhammed Alrezqi

**Affiliations:** 1 Family Medicine, King Abdulaziz Medical City, Ministry of National Guard Health Affairs, Jeddah, SAU; 2 Family Medicine, King Abdullah International Medical Research Center, Jeddah, SAU; 3 College of Medicine, King Saud bin Abdulaziz University for Health Sciences, Jeddah, SAU; 4 Primary Health Care, Ministry of National Guard Health Affairs, Jeddah, SAU; 5 Pharmacy, King Saud bin Abdulaziz University for Health Sciences, Jeddah, SAU

**Keywords:** cardiovascular risk, chronic kidney disease, dapagliflozin, glp-1 receptor agonist, glycemic control, hba1c, real-world study, semaglutide, sglt2 inhibitor, type 2 diabetes mellitus

## Abstract

Background: Combination therapy with glucagon-like peptide-1 receptor agonists and sodium-glucose cotransporter-2 inhibitors may provide complementary metabolic, cardiovascular, and renal benefits in type 2 diabetes mellitus (T2DM). However, real-world evidence from Middle Eastern populations remains limited. This study evaluated the effectiveness and safety of combined semaglutide and dapagliflozin therapy in routine clinical practice.

Methods: This retrospective cohort study included adults (≥18 years) with T2DM treated with concurrent semaglutide and dapagliflozin for at least 12 months at a specialized primary care clinic in King Abdulaziz Medical City, Jeddah, Saudi Arabia. Clinical and laboratory data were extracted from electronic medical records. The primary outcome was a change in glycated hemoglobin (HbA1c). Secondary outcomes included changes in the lipid profile, renal parameters (estimated glomerular filtration rate (eGFR) and urinary albumin-to-creatinine ratio (UACR)), and adverse events. Paired comparisons were performed using the Wilcoxon signed-rank test. Multivariable linear regression was used to identify predictors of HbA1c reduction.

Results: A total of 291 patients were included (mean age 58.6 ± 9.5 years; 49.8% male; mean diabetes duration 12.9 ± 9.5 years). Median HbA1c decreased from 8.20% to 7.20% (Z = −10.323; P < 0.001). Significant reductions were also observed in total cholesterol, LDL cholesterol, and triglycerides (all P < 0.001), while the UACR decreased significantly (P < 0.001). eGFR remained stable (P = 0.376). Most patients (90.4%) experienced no documented adverse events; hypoglycemia occurred in 6.2%. In multivariable analysis, higher baseline HbA1c (B = 0.552; P < 0.001), hypertension (B = 0.413; P = 0.016), and dyslipidemia (B = 0.381; P = 0.021) were associated with greater HbA1c reduction, whereas insulin use was associated with a smaller reduction (B = −0.612; P < 0.001). The model explained 43.2% of the variability in HbA1c change.

Conclusions: In routine clinical practice, combined semaglutide and dapagliflozin therapy was associated with significant improvements in glycemic control, lipid profile, and albuminuria, with stable renal function and a favorable safety profile. These findings support the effectiveness of this combination in real-world settings and highlight the need for prospective comparative studies to confirm long-term outcomes and define optimal patient selection.

## Introduction

Type 2 diabetes mellitus (T2DM) is a progressive metabolic disorder characterized by chronic hyperglycemia resulting from insulin resistance and progressive β-cell dysfunction [1,2]. Despite advances in pharmacotherapy, many patients fail to achieve adequate glycemic control and remain at increased risk for cardiovascular disease, chronic kidney disease, and premature mortality [2-4]. As the global prevalence of diabetes continues to rise, contemporary management has shifted beyond glycemic control toward therapies that also improve cardiovascular, renal, and metabolic outcomes [1-4].

Current international guidelines recommend glucagon-like peptide-1 receptor agonists (GLP-1 RAs) and sodium-glucose cotransporter-2 inhibitors (SGLT2is) as preferred therapeutic agents for patients with T2DM who have obesity, established cardiovascular disease, heart failure, or chronic kidney disease [1,5]. These agents provide clinically meaningful reductions in glycated hemoglobin (HbA1c), body weight, and cardiometabolic risk while demonstrating favorable cardiovascular and renal protective effects independent of glucose lowering [2,4].

Semaglutide, a long-acting GLP-1RA, improves glycemic control by enhancing glucose-dependent insulin secretion, suppressing glucagon release, delaying gastric emptying, and reducing appetite, thereby promoting substantial weight loss [1-6]. Large randomized clinical trials have demonstrated significant reductions in HbA1c, body weight, and major adverse cardiovascular events among patients receiving semaglutide [2,3,5]. Similarly, dapagliflozin, an SGLT2i, lowers plasma glucose by promoting urinary glucose excretion and has consistently demonstrated benefits in reducing hospitalization for heart failure, slowing the progression of chronic kidney disease, and improving cardiovascular outcomes [3-9].

Because GLP-1 RAs and SGLT2is target complementary pathophysiological mechanisms involved in T2DM, their combined use has attracted considerable clinical interest. According to DeFronzo's "ominous octet" model, T2DM results from multiple interacting metabolic defects rather than isolated insulin resistance or β-cell dysfunction. Combination therapy simultaneously addresses several of these abnormalities by improving insulin secretion, reducing glucotoxicity, promoting weight loss, and enhancing insulin sensitivity, thereby providing a strong biological rationale for additive or synergistic therapeutic effects [6-10].

Emerging evidence from randomized trials and real-world observational studies suggests that combining semaglutide with dapagliflozin may produce greater improvements in glycemic control and body weight than either agent alone. In addition, combination therapy has been associated with favorable effects on lipid profiles, blood pressure, albuminuria, and renal function while maintaining an acceptable safety profile. However, much of the available evidence has been generated from clinical trials conducted in highly selected populations or from observational studies in Europe and North America [3-14]. Consequently, the effectiveness of this therapeutic strategy in routine clinical practice within Middle Eastern populations remains insufficiently characterized.

Real-world evidence is particularly important because patients encountered in routine clinical practice often differ substantially from participants enrolled in randomized controlled trials with respect to age, disease duration, comorbidities, concomitant medications, and treatment adherence. Evaluating treatment effectiveness in these settings provides complementary information regarding the generalizability of clinical trial findings and helps inform clinical decision-making.

To date, there is limited evidence from Saudi Arabia and the Gulf region evaluating the effectiveness of combined semaglutide and dapagliflozin therapy in patients with T2DM. Therefore, this retrospective cohort study was conducted to assess changes in glycemic control, lipid profile, renal function, and treatment safety among adults with T2DM receiving concurrent semaglutide and dapagliflozin therapy in a specialized primary care diabetes clinic. Additionally, we sought to identify clinical factors associated with greater improvement in glycemic control following combination therapy.

## Materials and methods

Study design and setting

This retrospective cohort study was conducted at the Specialized Polyclinic, Primary Health Care Center, King Abdulaziz Medical City (KAMC), Jeddah, Saudi Arabia, under the Ministry of National Guard Health Affairs. The study evaluated the real-world effectiveness of combined semaglutide and dapagliflozin therapy in adults with T2DM using routinely collected electronic medical records (EMRs).

Study population

Adult patients (≥18 years) with an established diagnosis of T2DM who received concurrent treatment with semaglutide and dapagliflozin for at least 12 months were eligible for inclusion. Patients were required to have complete baseline and follow-up clinical and laboratory data, including HbA1c, body weight, body mass index (BMI), lipid profile, estimated glomerular filtration rate (eGFR), and urinary albumin-to-creatinine ratio (UACR), where available.

Patients with type 1 diabetes mellitus or secondary diabetes, severe renal impairment (eGFR <30 mL/min/1.73 m²), end-stage kidney disease, pregnancy during the study period, active malignancy, or incomplete records for the primary study outcomes were excluded.

Sample size and sampling

The minimum required sample size was estimated using the Raosoft sample size calculator [15], assuming a source population of approximately 1,200 patients, a 95% confidence level, a 5% margin of error, and a response distribution of 50%. The calculated minimum sample size was 291 participants. A convenience sampling strategy was employed whereby all eligible patients meeting the inclusion criteria during the study period were included. The final study cohort comprised 291 patients.

Data collection

Data were extracted retrospectively from the institutional EMR system using a standardized data collection form. Extracted variables included demographic characteristics (age and sex), diabetes duration, diabetes type, comorbidities, concomitant medications, anthropometric measurements, glycemic indices, lipid profile, renal function, and documented adverse events.

Baseline variables included body weight, BMI, HbA1c, total cholesterol, low-density lipoprotein (LDL) cholesterol, high-density lipoprotein (HDL) cholesterol, triglycerides, eGFR, and UACR. Follow-up values for the same parameters were collected after at least 12 months of combined semaglutide and dapagliflozin therapy.

Comorbidities recorded included hypertension, dyslipidemia, chronic kidney disease, cardiovascular disease, obesity, chronic obstructive pulmonary disease, asthma, thyroid disorders, and malignancy. Concomitant antidiabetic and cardiovascular medications, including metformin, insulin, sulfonylureas, dipeptidyl peptidase-4 (DPP-4) inhibitors, statins, angiotensin-converting enzyme inhibitors/angiotensin receptor blockers (ACE inhibitors/ARBs), beta-blockers, and aspirin, were also documented.

The primary outcome was the change in HbA1c following combination therapy. Secondary outcomes included changes in lipid profile, renal function (eGFR and UACR), and the occurrence of adverse events during treatment.

Statistical analysis

Statistical analyses were performed using IBM SPSS Statistics for Windows, Version 26 (Released 2018; IBM Corp., Armonk, New York, United States). Continuous variables were initially assessed for normality using the Shapiro-Wilk test and visual inspection of histograms and normal probability plots. As the distributions of the paired outcome variables deviated from normality, continuous variables are presented as medians with corresponding measures of central tendency in the tables, and paired comparisons between baseline and follow-up measurements were performed using the Wilcoxon signed-rank test.

Normally distributed baseline descriptive variables are reported as mean ± standard deviation (SD), whereas categorical variables are presented as frequencies and percentages.

To identify independent predictors of glycemic improvement, a multivariable linear regression model was constructed using the change in HbA1c (baseline minus follow-up HbA1c) as the dependent variable. Candidate independent variables were selected a priori based on their clinical relevance and previous literature and included age, duration of diabetes, baseline body weight, baseline HbA1c, hypertension, dyslipidemia, chronic kidney disease, cardiovascular disease, metformin use, and insulin use. Regression coefficients (B), standardized beta coefficients (β), 95% confidence intervals (CIs), and corresponding P values were reported. Overall model performance was assessed using the coefficient of determination (R²), adjusted R², and the overall F statistic. All statistical tests were two-sided, and a P-value <0.05 was considered statistically significant.

## Results

Baseline characteristics

A total of 291 patients receiving combined semaglutide and dapagliflozin therapy were included in the analysis. The mean age was 58.6 ± 9.5 years, and 145 (49.8%) participants were male. The mean duration of diabetes was 12.9 ± 9.5 years among the 287 patients for whom disease duration was available. Nearly all participants had type 2 diabetes mellitus (288 (99.0%)), whereas two patients (0.7%) had type 1 diabetes and one (0.3%) had latent autoimmune diabetes in adults (LADA) (Table 1).

**Table 1:** Baseline demographic and clinical characteristics of the study population (N = 291) BMI, body mass index; HbA1c, glycated hemoglobin; LDL, low-density lipoprotein; HDL, high-density lipoprotein; eGFR, estimated glomerular filtration rate; UACR, urinary albumin-to-creatinine ratio; COPD, chronic obstructive pulmonary disease; ACE, angiotensin-converting enzyme; ARB, angiotensin receptor blocker.

Characteristic		Value
Demographic characteristics	Age (years), mean ± SD	58.6 ± 9.5
	Male sex, n (%)	145 (49.8)
	Duration of diabetes (years), mean ± SD	12.9 ± 9.5 (n = 287)
Type of diabetes, n (%)	Type 2 diabetes	288 (99.0)
	Type 1 diabetes	2 (0.7)
	Latent autoimmune diabetes in adults (LADA)	1 (0.3)
Baseline clinical characteristics	Weight (kg), mean ± SD	88.5 ± 17.8
	Body mass index (kg/m²), mean ± SD	33.8 ± 7.7 (n = 290)
	HbA1c (%), mean ± SD	8.4 ± 1.9 (n = 290)
Baseline laboratory parameters	Total cholesterol (mmol/L), mean ± SD	4.42 ± 1.19
	LDL cholesterol (mmol/L), mean ± SD	2.63 ± 1.06 (n = 290)
	HDL cholesterol (mmol/L), mean ± SD	1.13 ± 0.68
	Triglycerides (mmol/L), mean ± SD	1.58 ± 0.99
	eGFR (mL/min/1.73 m²), mean ± SD	93.3 ± 19.8
	UACR (mg/g), median	3.65 (n = 136)
Comorbidities, n (%)	Hypertension	193 (66.3)
	Dyslipidemia	112 (38.5)
	Cardiovascular disease	43 (14.8)
	Obesity	18 (6.2)
	Chronic kidney disease	11 (3.8)
	COPD	2 (0.7)
	Cancer	2 (0.7)
Concomitant medications, n (%)	Metformin	236 (81.1)
	Insulin	136 (46.7)
	Sulfonylurea	104 (35.7)
	DPP-4 inhibitor	53 (18.2)
	Statin	32 (11.0)
	ACE inhibitor/ARB	17 (5.8)
	Aspirin	7 (2.4)
	Beta-blocker	4 (1.4)

At baseline, the mean body weight was 88.5 ± 17.8 kg, and the mean body mass index (BMI) was 33.8 ± 7.7 kg/m². The mean baseline HbA1c was 8.4 ± 1.9%. Baseline laboratory parameters included a mean total cholesterol concentration of 4.42 ± 1.19 mmol/L, LDL cholesterol of 2.63 ± 1.06 mmol/L, HDL cholesterol of 1.13 ± 0.68 mmol/L, triglycerides of 1.58 ± 0.99 mmol/L, and estimated glomerular filtration rate (eGFR) of 93.3 ± 19.8 mL/min/1.73 m². Among the 136 patients with available UACR measurements, the median baseline UACR was 3.65 mg/g.

Hypertension was the most common comorbidity, affecting 193 patients (66.3%), followed by dyslipidemia in 112 (38.5%) and cardiovascular disease in 43 (14.8%). Chronic kidney disease was present in 11 patients (3.8%). Regarding concomitant medications, metformin was prescribed in 236 patients (81.1%), insulin in 136 patients (46.7%), sulfonylureas in 104 patients (35.7%), and DPP-4 inhibitors in 53 patients (18.2%) (Table 1).

Changes in metabolic and laboratory parameters

Following treatment with combined semaglutide and dapagliflozin therapy, significant improvements were observed in multiple metabolic parameters (Table 2). Median HbA1c decreased from 8.20% at baseline to 7.20% at follow-up (Wilcoxon Z = −10.323, P < 0.001). Significant reductions were also observed in total cholesterol (4.30 vs. 4.05 mmol/L; Z = −5.269, P < 0.001), LDL cholesterol (2.50 vs. 2.19 mmol/L; Z = −5.467, P < 0.001), and triglycerides (1.38 vs. 1.20 mmol/L; Z = −3.646, P < 0.001). Although the median HDL cholesterol remained unchanged at 1.10 mmol/L, the paired comparison demonstrated a statistically significant increase (Z = −3.503, P < 0.001).

**Table 2:** Changes in clinical and laboratory parameters following combined semaglutide and dapagliflozin therapy HbA1c, glycated hemoglobin; LDL, low-density lipoprotein; HDL, high-density lipoprotein; eGFR, estimated glomerular filtration rate; UACR, urinary albumin-to-creatinine ratio.

Variable	Baseline median	Follow-up median	Wilcoxon Z	P-value
HbA1c (%)	8.20	7.20	-10.323	<0.001
Total cholesterol (mmol/L)	4.30	4.05	-5.269	<0.001
LDL cholesterol (mmol/L)	2.50	2.19	-5.467	<0.001
HDL cholesterol (mmol/L)	1.10	1.10	-3.503	<0.001
Triglycerides (mmol/L)	1.38	1.20	-3.646	<0.001
eGFR (mL/min/1.73 m²)	98.0	99.5	-0.885	0.376
UACR (mg/g)	3.65	2.25	-3.910	<0.001

Renal parameters showed mixed findings. The median UACR decreased significantly from 3.65 to 2.25 mg/g (Z = −3.910, P < 0.001), whereas no significant change was observed in eGFR, with median values of 98.0 and 99.5 mL/min/1.73 m² at baseline and follow-up, respectively (Z = −0.885, P = 0.376).

Adverse events

Overall, combined therapy was well tolerated. No adverse events were documented in 263 patients (90.4%). Hypoglycemia was the most frequently reported adverse event, occurring in 18 patients (6.2%), followed by gastrointestinal symptoms in eight patients (2.7%). Two additional adverse events (0.7%) were reported, including one urinary tract infection and one unspecified semaglutide-related adverse event (Table 3).

**Table 3:** Adverse events reported during combined semaglutide and dapagliflozin therapy (N = 291)

Adverse event	n (%)
No adverse events	263 (90.4)
Hypoglycemia	18 (6.2)
Gastrointestinal symptoms	8 (2.7)
Other adverse events	2 (0.7)

Predictors of HbA1c reduction

Multivariable linear regression identified baseline HbA1c, hypertension, dyslipidemia, and insulin use as independent predictors of HbA1c reduction following combined semaglutide and dapagliflozin therapy (Table 4). Higher baseline HbA1c was the strongest predictor of greater HbA1c reduction (β = 0.646; B = 0.552, 95% CI, 0.472 to 0.631; P < 0.001). Hypertension (B = 0.413, 95% CI, 0.078 to 0.748; β = 0.119; P = 0.016) and dyslipidemia (B = 0.381, 95% CI, 0.057 to 0.704; β = 0.112; P = 0.021) were also independently associated with greater reductions in HbA1c. Conversely, insulin use was independently associated with a smaller HbA1c reduction (B = −0.612, 95% CI, −0.936 to −0.287; β = −0.185; P < 0.001). Age, duration of diabetes, baseline body weight, chronic kidney disease, cardiovascular disease, and metformin use were not significantly associated with changes in HbA1c.

**Table 4:** Multivariable linear regression analysis of factors associated with HbA1c reduction following combined semaglutide and dapagliflozin therapy Model performance: R² = 0.432; adjusted R² = 0.412; F(10,274) = 20.879; P < 0.001. HbA1c, glycated hemoglobin; CI, confidence interval.

Predictor	Regression coefficient, B (95% CI)	Standardized β	P-value
Age (years)	-0.004 (-0.020 to 0.013)	-0.021	0.674
Duration of diabetes (years)	-0.012 (-0.030 to 0.006)	-0.068	0.194
Baseline weight (kg)	0.001 (-0.007 to 0.010)	0.013	0.775
Baseline HbA1c (%)	0.552 (0.472 to 0.631)	0.646	<0.001
Hypertension	0.413 (0.078 to 0.748)	0.119	0.016
Dyslipidemia	0.381 (0.057 to 0.704)	0.112	0.021
Chronic kidney disease	-0.093 (-0.883 to 0.697)	-0.011	0.817
Cardiovascular disease	0.329 (-0.124 to 0.782)	0.068	0.154
Metformin use	-0.173 (-0.565 to 0.219)	-0.041	0.387
Insulin use	-0.612 (-0.936 to -0.287)	-0.185	<0.001

The final regression model explained 43.2% of the variability in HbA1c reduction (R² = 0.432; adjusted R² = 0.412) and demonstrated good overall model fit (F = 20.879, P < 0.001).

## Discussion

In this retrospective cohort study of adults with diabetes receiving combined semaglutide and dapagliflozin therapy in routine clinical practice, we observed significant improvements in glycemic control, lipid profile, and albuminuria over the study period. HbA1c decreased significantly after initiation of combination therapy, accompanied by reductions in total cholesterol, LDL cholesterol, triglycerides, and UACR. Renal filtration function, as measured by eGFR, remained stable. Overall, treatment was well tolerated, with most patients experiencing no documented adverse events. In multivariable analysis, higher baseline HbA1c, hypertension, and dyslipidemia were independently associated with greater HbA1c reduction, whereas concurrent insulin use was associated with a smaller improvement.

The observed reduction in HbA1c is consistent with the complementary mechanisms of action of GLP-1 RAs and SGLT2is. Semaglutide enhances glucose-dependent insulin secretion, suppresses glucagon secretion, delays gastric emptying, and reduces appetite, whereas dapagliflozin lowers plasma glucose through urinary glucose excretion while reducing glucotoxicity. Acting through distinct yet complementary pathways, these agents target several components of the pathophysiology of type 2 diabetes, providing a biologically plausible explanation for the glycemic improvements observed in our cohort [2-9].

Our findings are consistent with previous randomized and real-world studies evaluating combined GLP-1 RA and SGLT2i therapy. Some studies demonstrated that the addition of semaglutide to ongoing SGLT2i treatment produced clinically meaningful reductions in HbA1c and body weight compared with placebo [3-7]. Similarly, observational studies have reported that combined semaglutide and dapagliflozin therapy results in greater improvements in glycemic control than monotherapy while maintaining an acceptable safety profile [7-11]. The present study extends these observations by providing real-world evidence from Saudi Arabia, a region in which data regarding this combination remain limited.

Beyond glycemic control, we observed significant improvements in multiple lipid parameters, including total cholesterol, LDL cholesterol, and triglycerides. Although the median HDL cholesterol remained unchanged, paired analysis demonstrated a statistically significant improvement, suggesting modest favorable effects on lipid metabolism. These findings are consistent with previous reports indicating that both GLP-1 RAs and SGLT2is exert beneficial metabolic effects beyond glucose lowering through reductions in adiposity, improvements in insulin sensitivity, and modulation of hepatic lipid metabolism. Such changes may contribute to the reduction in long-term cardiovascular risk observed in large cardiovascular outcome trials [4-9].

Renal outcomes also demonstrated potentially favorable findings. Although no significant change was observed in the eGFR during follow-up, the UACR decreased significantly, suggesting improvement in albuminuria. Preservation of eGFR over the study period is itself clinically reassuring, particularly in a population with longstanding diabetes, where progressive decline in renal function would otherwise be anticipated. These findings are consistent with previous evidence demonstrating that SGLT2is primarily slow the rate of kidney function decline while reducing albuminuria, rather than producing large short-term increases in eGFR [7-14].

Treatment was generally well tolerated. More than 90% of patients had no documented adverse events, while hypoglycemia and gastrointestinal symptoms were relatively uncommon. Gastrointestinal symptoms are well-recognized adverse effects of GLP-1 RAs, whereas hypoglycemia is generally uncommon with either semaglutide or dapagliflozin alone but may occur in patients receiving concomitant insulin therapy. The low frequency of reported adverse events in our study supports the overall safety profile of combination therapy in routine clinical practice. However, underreporting inherent to retrospective studies should be considered.

An important finding of this study was that baseline HbA1c was the strongest independent predictor of glycemic improvement. Patients with poorer glycemic control before treatment experienced greater HbA1c reductions, a finding that has been consistently reported across clinical trials evaluating both GLP-1 RAs and SGLT2is [2-8]. Hypertension and dyslipidemia were also independently associated with greater HbA1c reduction. However, the biological explanation for these associations remains uncertain and may reflect differences in disease phenotype, treatment intensity, or overall cardiovascular risk. Conversely, concurrent insulin therapy was associated with a smaller HbA1c reduction, possibly reflecting more advanced diabetes, greater β-cell dysfunction, or increased disease severity among insulin-treated patients [5-10].

The present study has several strengths. It represents one of the few real-world evaluations of combined semaglutide and dapagliflozin therapy from Saudi Arabia and includes a relatively large cohort of patients managed in routine clinical practice. The availability of paired clinical and laboratory measurements allowed comprehensive assessment of glycemic, metabolic, and renal outcomes following treatment. Furthermore, multivariable regression analysis enabled adjustment for several clinically relevant confounding factors when evaluating predictors of glycemic response.

Several limitations should also be acknowledged. First, the retrospective observational design precludes causal inference and is subject to residual confounding. Second, this was a single-center study, which may limit the generalizability of the findings to other healthcare settings or populations. Third, the absence of a comparison group receiving either monotherapy or alternative glucose-lowering treatment prevents direct assessment of the incremental benefit attributable to combination therapy. Fourth, several variables, particularly UACR, contained substantial missing data, reducing the effective sample size for some analyses. Finally, medication adherence, lifestyle modifications, dietary factors, and treatment intensification during follow-up could not be fully assessed and may have influenced the observed outcomes.

## Conclusions

In this retrospective cohort study, combined semaglutide and dapagliflozin therapy was associated with significant improvements in glycemic control, lipid profile, and albuminuria among adults with diabetes managed in a real-world primary care setting, while renal function remained stable and the incidence of adverse events was low. Greater reductions in HbA1c were independently associated with higher baseline HbA1c levels, whereas concurrent insulin therapy was associated with a smaller glycemic response. These findings support the effectiveness and tolerability of combined semaglutide and dapagliflozin therapy in routine clinical practice and provide valuable real-world evidence from Saudi Arabia, where such data remain limited. Prospective multicenter studies with longer follow-up and appropriate comparator groups are warranted to confirm these findings and further define the patients most likely to benefit from this therapeutic approach.
